
JOURNAL INFORMATION
==============================
NLM Title Abbreviation: Inter Econ
Journal ID: Inter Econ
NLM Journal ID: 101086399

Inter Economics

ISSN: 0020-5346

ARTICLE INFORMATION
==============================
PMCID: PMC7110260
PMID: 32287363
DOI: 10.1007/s10272-020-0871-7
Article ID: 871
Article version: 1
Subjects: Editorial

The Pandemic Requires a Coordinated Global Economic Response

Herrero Alicia García alicia.garcia-herrero@bruegel.org

grid.432713.0 Bruegel, Rue de la Charité 33, 1210 Brussels, Belgium
Electronic publication date: 2020 Apr 1
Print publication date: 2020
Volume: 55
Issue: 2
First page: 66
Last page: 67
Copyright: © The Author(s) 2020
License: Open Access This article is licensed under a Creative Commons Attribution 4.0 International License, which permits use, sharing, adaptation, distribution and reproduction in any medium or format, as long as you give appropriate credit to the original author(s) and the source, provide a link to the Creative Commons licence, and indicate if changes were made.
The images or other third party material in this article are included in the article’s Creative Commons licence, unless indicated otherwise in a credit line to the material. If material is not included in the article’s Creative Commons licence and your intended use is not permitted by statutory regulation or exceeds the permitted use, you will need to obtain permission directly from the copyright holder.
To view a copy of this licence, visit http://creativecommons.org/licenses/by/4.0/.
License URL: https://creativecommons.org/licenses/by/4.0/

issue-copyright-statement: © The Author(s) 2020

==============================

Global pandemics are costly for many reasons. First and foremost, for their death toll and, more generally, for health and social reasons. The second reason is economic. I will focus on the latter given my field of expertise.

The economic impact of a pandemic depends upon the way it is tackled both from a health and an economic perspective. Regarding the former, strategies to deal with pandemics are mainly twofold, with two different economic outcomes. The first, mitigation, focuses on slowing but not necessarily stopping the spread of the virus, with the objective of reducing peak healthcare demand while protecting those most at risk. The second, suppression, aims at reversing the epidemic growth but with the risk of a rebound any time the suppression measures are lifted. The first is bound to last longer but have less intense social and economic consequences. The second should be faster but also carries extreme economic consequences, not only due to vanishing demand but also collapsing supply. The hope, though, is that the economy can recover more quickly as long as there is no pick up in the number of cases. It should be noted that a pandemic tends to have a timeframe of 18 months, until a vaccine becomes available.

Before moving to the economic policy responses to the pandemic depending on the disease containment response given (mitigation or suppression), it seems important to understand how different the economic consequences may be. Mitigation, being a longer but less abrupt strategy, should have a negative impact on demand but less so on supply as the population will not be fully locked down. In other words, depression (deflation and recession) is the most likely economic outcome. If a government chooses to supress the pandemic, as China did, especially in Hubei province, the supply shock might be greater, as most tasks requiring physical presence cannot be performed in a full lockdown. Within that context, stagflation (high inflation and recession) is the most likely scenario.

The response to depression is well known as the Keynesian policies that were introduced after the Great Depression were widely reviewed in standard economic textbooks. Massive fiscal stimulus is the key with the help of central banks and international policy coordination to avoid beggar-thy-neighbour policies. In the case of stagflation, central banks have their hands tied due to inflation pressures. Most importantly, supply-side policies are needed to increase the supply. While these differences in policies are relevant, it seems clear to me that the overall global shock is depression as panic in financial markets has contributed to the lack of demand from consumers and investment plans by corporations. More importantly, the world is much more integrated than in the late 1920s at the time of the Great Depression, which means that international cooperation is absolutely essential to address the hugely negative economic impact of a pandemic.

While international cooperation is, by nature, necessary during a global shock like a pandemic, policymakers have increasingly been unwilling to cooperate on global issues. A number of examples come to mind, from the demise of the World Trade Organization to the US-led trade war against China and, to a lesser extent, against the European Union. International policy cooperation is needed to increase the effectiveness of expansionary monetary and fiscal policies carried out at the national level and to avoid beggar-thy-neighbour policies, such as competitive devaluations. Beyond its general usefulness, international policy cooperation is even more essential than in 2008 for a number of reasons. First, the shock to the real economy is bound to be bigger than that of the global financial crisis given the number of countries directly affected. Second, it hits the heart of the real economy, namely companies' cashflows as well as households' income. This damage will soon be extended through companies' inability to repay banks' balance sheets, worsening asset quality and solvency at a time when banks' regulatory constraints are much tougher than in 2008. Third, the non-banking part of the financial system has become huge and lacks direct access to central bank liquidity. Fourth, the global financial system is even more interrelated than in the past. In fact, the stock of foreign direct investment and portfolio flows had continued to increase and we are now seeing huge outflows from emerging economies. Finally, risky assets are a bigger asset class than in 2008, not only in the developed world with the huge surge in high yield credit but also those from emerging and frontier economies.

The reality is that no single central bank or government alone can pacify markets and get the crisis under control. In fact, if economic leaders continue to act at a national level, as has been the case for the past few weeks, there is a risk of a race to the bottom and beggar-thy-neighbour policies. This goes beyond the usual financial variables all the way to medical equipment – which is also uncoordinated. Export controls of necessary medical supplies are to be avoided. Leaders should discuss how to pool their capacities and work together to stem the health crisis. But beyond that, bold economic steps are needed. Within that context, here are some takes of the key aspects needed as regards monetary cooperation.

First, monetary policy coordination must go beyond a series of rate cuts and quantitative programs by individual central banks. There is an increasing shortage of US dollars in international financial markets, which was one of the key reasons for the defaults of major financial institutions in 2008. To prevent the current pandemic from becoming a full-fledged global financial crisis, dollar liquidity needs to be granted cross-border. To that end, the existing swap lines that the US Federal Reserve has kept with major central banks since the global financial crisis (European Central Bank, Bank of Japan, Bank of Canada, Bank of England and Swiss National Bank) need to be expanded and made even cheaper and longer in maturity. In addition, central banks in the emerging world desperately need the dollar liquidity that the Fed or the International Monetary Fund will need to extend.

Second, dislocations in foreign exchange markets are increasing the odds of the collapse of a major financial institution – harkening back to 2008. Furthermore, large swings in oil markets only make this situation more dangerous. In particular, financial institutions outside the G7 are vulnerable since they do not have access to the same kind of refinancing facilities but desperately need hard currency. All in all, coordinated forex intervention should be high on the agenda of international policy coordination. This will also help avoid beggar-thy-neighbour actions.

Third, fiscal policy is key to providing liquidity to corporates and households in a scenario of depression, as Keynes pointed out. A coordinated fiscal stimulus is needed to reduce the risk of free riding. It is crucial therefore that national fiscal authorities – in a coordinated manner – provide much-needed relief to vulnerable individuals and the entire corporate sector, for example, by taking over part of the social security payments that companies pay, as well as mortgages for households. In situations like this, it is best that the government insures the economy. The cost of that insurance will be passed to all tax payers later on in one way or another; however, this is not the time to discuss the end of the crisis but how to end it.

All in all, the COVID-19 pandemic is a symmetric global shock that equates to vanishing demand globally – with instances of a negative supply shock and, thus, shortages, for some sectors, especially in countries following suppression strategies. Such shock requires fiscal and monetary stimulus in a coordinated manner. There is no time to lose.

Open Access funding provided by ZBW — Leibniz Information Centre for Economics.

Alicia García Herrero, Bruegel, Brussels, Belgium.

